# Individualistic Versus Collaborative Learning in an eHealth Literacy Intervention for Older Adults: Quasi-Experimental Study

**DOI:** 10.2196/41809

**Published:** 2023-02-09

**Authors:** Christian Elias Vazquez, Bo Xie, Kristina Shiroma, Neil Charness

**Affiliations:** 1 School of Social Work The University of Texas at Arlington Arlington, TX United States; 2 School of Nursing The University of Texas at Austin Austin, TX United States; 3 School of Information The University of Texas at Austin Austin, TX United States; 4 Department of Psychology Florida State University Tallahassee, FL United States

**Keywords:** eHealth literacy, digital literacy, older adults, eHealth, aging, web-based information, health information

## Abstract

**Background:**

Older adults tend to have insufficient health literacy, which includes eHealth literacy—the ability to access, assess, and use digital health information. Interventions using methods such as collaborative learning (CL) and individualistic learning (IL) may be effective in addressing older adults’ low eHealth literacy, but little is known about the short- and long-term effects of CL versus IL on older adults’ eHealth literacy.

**Objective:**

The objective of this study was to use a 3 × 2 × 3 mixed factorial design to examine older adults’ learning with CL versus IL for eHealth literacy.

**Methods:**

Older adults (N=466; mean age 70.5, SD 7.2; range 60-96 years) from diverse racial and ethnic groups were randomly assigned to either the CL or IL group (233/466, 50% in each). The intervention consisted of 4 weeks of training in 2-hour sessions held twice a week. Using ANOVA and multiple regression, we focused on the main effects of learning condition and interaction between learning condition and previous computer experience. Learning method (CL or IL) and previous computer experience (experienced, new, or mixed) were between-subject variables, and time of measurement (pretest measurement, posttest measurement, and 6-month follow-up) was the within-subject variable. Primary outcome variables were eHealth literacy efficacy, computer and web knowledge, basic computer and web operation skills, information-seeking skills, and website evaluation skills. Control variables were age, sex, education, health status, race and ethnicity, income, primary language, and previous health literacy.

**Results:**

eHealth literacy efficacy, computer and web knowledge, basic computer and web operation skills, information-seeking skills, and website evaluation skills improved significantly (*P*<.001 in all cases) from before to after the intervention. From postintervention measurement to 6-month follow-up, there was a significant interaction between learning condition and previous computer experience based on 1 outcome measure, computer and web operation skills (*F*_2,55_=3.69; *P*=.03). To maintain computer and web operation skills 6 months after the intervention, it was more effective for people with little to no previous computer experience to learn individually, whereas for people with more previous computer experience, it was more effective to learn collaboratively. From postintervention measurement to 6-month follow-up, statistically significant decreases were found in 3 of the 5 outcome measures: eHealth literacy efficacy, computer and web knowledge, and basic computer and web operation skills (*P*<.001 for all 3 cases).

**Conclusions:**

Older adults’ eHealth literacy can be improved through effective intervention, and the IL or CL condition may have little effect on short-term outcomes. However, to maintain long-term benefits, it may be best to learn collaboratively with others who have similar previous computer experience. eHealth literacy is multidimensional, with some components retained better over time. Findings suggest a need for resources to provide continuous training or periodic boosting to maintain intervention gains.

## Introduction

### Background

Health literacy is defined as the degree to which individuals have the capacity to obtain, process, and understand the basic health information and services needed to make appropriate health decisions [1]. Health literacy is important as those with below-basic levels of health literacy are at greater risk of lagging their peers across several health outcomes [1]. Older age is strongly associated with lower health literacy [2]. In fact, of all adult groups in the United States, the older adult population has the lowest health literacy level—a critical issue given the rapidly growing US older adult population [3-5]. Older adults often have multiple chronic health conditions that increase their interactions with health care providers, which in turn increases their need for sufficient health literacy [6]. However, only 3% of older adults in the United States have proficient health literacy [4].

Researchers continue to assess which components are critical for effective health literacy interventions and implementation strategies for older adults. A systematic review by Walters et al [7] highlighted the increasing attention of research on health literacy interventions in recent years, with just 5 studies published before 2017 and 17 studies published up to the first quarter of 2020. This review reinforces findings from various earlier reviews showing that few interventions have determined best practices for health literacy interventions, such as whether older adults learn better in groups or as individuals [7-12].

### eHealth Literacy

Health literacy research continues to evolve as the conceptualization of health literacy evolves. As information and communication technologies become integral in delivering and receiving health care, areas such as eHealth literacy have emerged [13]. In 2006, Norman and Skinner [14] promoted the concept of eHealth literacy as “the ability to seek, find, understand, and appraise health information from electronic sources and apply the knowledge gained to addressing or solving a health problem.” Norman [15] further pointed out that, as technology changes, so do the requirements for eHealth literacy skills. Health agencies such as the Centers for Disease Control and Prevention are increasingly providing health information on the web, making the internet an important and sometimes the main source of health information accessed via mobile phones and tablets [16].

This shift to electronic dissemination of health information has implications for health literacy interventions, suggesting that they should focus on eHealth literacy [17]. Such a focus is important as there is some evidence suggesting that older adults are interested in seeking health information on the web [18], yet older adults tend to have low digital literacy [19]. This can be addressed via eHealth literacy interventions that increase digital skills [20]. To address eHealth literacy challenges, it is essential to conceptualize health literacy as an active, dynamic process of lifelong learning [21], a process that goes beyond formal educational settings in early life and features continuous learning of new ways to find valid, reliable health information from trusted web-based resources [22]. This study is part of a series of projects that add to the health literacy literature by investigating the effectiveness of a theory-based intervention to contribute to the understanding of how different intervention strategies may affect older adults’ acquisition of eHealth literacy.

### Collaborative Learning in Community Settings

Collaborative learning (CL) refers to “any instructional method in which students work together in small groups toward a common goal” [23]. CL promotes engagement for both the individual and the group as students progress through the learning process [24]. Older adults value CL with and from their peers about important health issues such as diabetes and cancer [25]. Within the context of learning to use computers, CL has been found to be effective for both learning outcomes and social development in older adults [26-28], although an earlier study [29] found that older adults performed similarly in computer learning regardless of learning individually or in pairs. In the context of eHealth literacy, CL enables opportunities to learn new health information and skills to access such information on the web [21]. Similarly, Ahmad et al [26] have suggested that CL allows older adult learners to participate with peers and interact effectively to learn digital technology.

Health-related community-based research meets older adults in their communities to provide interventions in informal settings [30]. CL for older adults typically occurs in such settings as opposed to the formal educational settings that are more typical for younger people. The Electronic Health Information for Lifelong Learners (eHiLL) studies use community settings to integrate existing public infrastructure and resources such as public libraries and senior centers [31,32]. Existing and authoritative internet health information resources developed by the National Institutes of Health (NIH) enable this type of work to be replicable and accessible to all. A systematic literature review by Kim and Xie [9] revealed that interventions combined with educational programs at the community level can encourage target groups to use web-based health resources. Affirming the importance of informal learning environments for older adults’ success in digital learning, a systematic review by Ahmad et al [26] found that, across study samples and settings, informal learning environments provided older adults with opportunities to share their experiences, options, and expectations with their peers, which encouraged them to learn.

### Lifelong Learning

This study is part of the eHiLL research program, which aims to generate scientific knowledge of optimal learning conditions and strategies that can effectively and efficiently improve older adults’ learning and use of eHealth applications [20,31,33-35]. Each eHiLL study builds on previous work to examine the effects of various learning conditions and strategies through the testing of hypotheses in rigorous theory-driven interventions. eHiLL studies are informed by social interdependence theory, which supports the superiority of CL over individualistic learning (IL) [36]. This theory emphasizes interdependence among group members by arguing that the group is a dynamic whole [36]. A meta-analysis of >300 studies has provided strong evidence that CL outperforms IL and competitive learning in postsecondary and professional settings [24]. However, less is known about CL’s effectiveness among older adult eHealth learners in informal learning settings when learning computer skills. This eHiLL study was designed to address these gaps in the literature.

### Earlier eHiLL Studies and Gaps in the Literature

The first eHiLL project was a pilot study (N=172) with 1 arm to assess the effectiveness of CL with no comparison [20]. This study found evidence to suggest that CL can be a useful method for improving older adults’ eHealth literacy when paired with key elements of computer learning in older adults. The findings also indicated that social interdependence theory could be generalized beyond the younger population and formal educational settings. In a second study (N=124), we used a 2 × 2 × 2 mixed factorial design with learning method (CL and IL) and presentation (visual only and visual plus auditory) as between-subject variables and time of measurement (pre- and postintervention measurement) as the within-subject variable. The intervention, regardless of the specific combination of learning method and information presentation, was effective in improving eHealth literacy from before to after the intervention [34]. In a third study (N=146), we used a 2 × 2 mixed factorial design with learning method (CL and IL) as the between-subject variable and time of measurement (pre- and posttest measurement) as the within-subject variable to focus on CL versus IL in a new sample [21]. As in the second study, regardless of the specific learning method used, the eHealth literacy intervention significantly improved knowledge, skills, and eHealth literacy efficacy from before to after the intervention. However, CL did not differ from IL in affecting learning outcomes, suggesting that the previous widely reported advantages of CL over IL may not be easily applicable to the older population in informal settings. In all 3 studies, we used the same web-based learning modules and study protocols. Possible reasons that might have contributed to a lack of support for the superiority of CL over IL included relatively small sample sizes, underdeveloped CL strategies, potential confounding effects of various group compositions (eg, those based on sex and previous computer experience), and no follow-ups to examine potential longitudinal effects (because of a lack of resources for these pilot studies).

These earlier eHiLL studies [20,21,34], along with other studies reviewed by Ahmad et al [31], provide evidence that CL is effective for older adult populations even when using digital technologies. However, major gaps remain to be addressed, particularly with regard to longitudinal effects [20,37]. A systematic literature review by Manafo and Wong [37] found that, of 9 studies on health literacy programs for older adults, only 2 had a follow-up period, and neither of those studies reported any long-term outcomes. In addition, previous studies have identified differences in approaches to group composition in CL for older adults such that a heterogeneous group composition has been found to facilitate more successful CL than a homogeneous group composition [38,39]. Several studies have found that CL works better with either a female- or male-sex majority than in groups with equal sex composition [40,41], as well as with same-sex groups as opposed to mixed ones [41,42]. There is also some evidence suggesting that, for CL in older adults, it may be advantageous to form homogeneous groups based on previous computer experience [43]. Research is needed to address the implications of these findings in the literature.

### This Study

In this study, we address the aforementioned gaps in the literature by (1) using a large randomized sample, (2) adding a 6-month follow-up to examine how gains might be maintained beyond the intervention period, (3) adding group composition based on previous computer experience as an independent variable to investigate the effects of group composition on learning outcomes, and (4) developing and implementing detailed instructions and procedures to ensure CL versus IL in the respective groups. Guided by social interdependence theory and our own previous eHiLL studies [20,21,34], in this study, we examined the research questions and hypotheses outlined in Textbox 1.

**Research questions and hypotheses for this study.**

**Research questions (RQs)**

- RQ 1
  What are the differences between the main effects of the intervention (collaborative learning [CL] vs individualistic learning [IL]) on older adults’ eHealth literacy from pre- to posttest measurement?
- RQ 2
  Do the effects of CL interact with those of heterogeneous versus homogeneous computer-experience group composition?
- RQ 3
  How are the effects of CL versus IL maintained beyond the training period?

**Hypotheses**

- Hypothesis 1
  CL will be more effective than IL in improving older adults’ eHealth literacy.
- Hypothesis 2
  In the CL condition, the heterogeneous group composition (mixed users) will be more effective than either homogeneous group composition (experienced user–only and new user–only).
- Hypothesis 3
  The effects of CL will be better maintained than those of IL.

## Methods

### Design

For this intervention, we used a 3 × 2 × 3 mixed factorial design, with group composition based on previous computer experience (experienced, new, and mixed) and learning method (IL and CL) as between-subject variables and time of measurement (preintervention measurement, postintervention measurement, and 6-month follow-up) as the within-subject variable.

### Sample and Recruitment

Recruitment included the posting and distribution of flyers at the research sites and surrounding locations (eg, in grocery stores) as well as advertising in the research sites’ newsletters and local newspapers. Recruitment continued until the target sample size was reached. The inclusion criteria were as follows: (1) age ≥60 years, (2) ability to go to and from a research site, (3) fluency in English, and (4) interest in learning about using computers to find health information. A total of 466 older adults aged 60 to 96 years participated (mean age 70.5, SD 7.2 years for all; 70.1, SD 6.7 years for the CL group; and 70.8, SD 7.6 years for the IL group).

### Research Sites

Data were collected from 8 research sites: 2 public libraries in the greater Washington, District of Columbia, area; and 1 public library, 3 senior activity centers, 1 recreation center, and 1 senior living facility in the greater Austin, Texas, area. These sites were selected as they (1) served a large population of older adults of diverse ethnicities and socioeconomic status; (2) provided free networked computers, space, and staff support to facilitate the study’s implementation; (3) were geographically convenient for potential research participants and the researchers; and (4) were accessible by car or public transportation, thus enabling us to reach a diverse range of older adults.

### Ethics Approval

Before the intervention, participants signed a consent form approved by the institutional review boards of the authors’ institutions, the University of Texas at Austin (2012-05-0049) and the University of Maryland (07-0264).

### Procedure

The intervention consisted of 8 two-hour sessions: 1 preintervention test (session 1), 1 postintervention test (session 8), and 6 training sessions (sessions 2-7). Participants met twice a week for 4 weeks to complete the intervention.

Participants were randomly assigned to either IL classes or CL classes, with a maximum of 8 participants per class. In each training session, participants in both classes first watched the tutorial twice with a 5-minute break in between; then, they were given a handout to perform practice activities. A facilitator was available in each training session to set up the equipment, distribute handouts, and provide immediate help whenever needed.

In the IL classes, participants wore headphones and worked on their computers during the entire intervention to avoid interaction with peers. At the beginning of each session, the facilitator stated specifically that students should work independently and avoid interacting with peers. The tutorial in IL classes also reminded participants to learn and perform the activities independently throughout the session. Participants were encouraged to ask the facilitator for help whenever they had any questions.

In CL classes, to encourage collaboration, we asked groups of 2 or 3 participants to share a computer by using a multiheadphone splitter during the entire intervention. In this way, all members in a group could proceed at the same pace, and different groups would not interfere with each other. At the beginning of each session, the facilitator stressed that students in each group should learn together and work with their peers to perform the practice activities. The tutorial for the CL classes provided clear instructions throughout the session to encourage collaboration—for example, by taking turns or with group discussions and reflections. Multimedia Appendices 1 to 6 provide examples of IL and CL instructions as shown on participants’ computer screens.

### Instructional Materials

The instructional materials consisted of a series of web-based interactive tutorials developed for this study. The curriculum in the tutorials was guided by “Helping Older Adults Search for Health Information Online: A Toolkit for Trainers,” developed by the National Institute on Aging of the NIH. This free toolkit [44] is designed to improve older adults’ ability to find health information on 2 NIH websites: NIHSeniorHealth and MedlinePlus. The toolkit contains 9 modules: module 1 focuses on computer and internet basics, modules 2 to 5 introduce NIHSeniorHealth, modules 6 to 8 introduce MedlinePlus, and module 9 focuses specifically on improving one’s ability to appraise health information. As the NIHSeniorHealth website was being retired at the time of this study, we adapted the content of modules 1 and 6 to 9 to make each module fit a 2-hour training session (Table 1). We then developed 10 web-based interactive tutorials based on the 5 learning modules using Adobe Captivate (Adobe Inc.): 5 for IL classes and 5 for CL classes. Specific instructions and activities were developed to ensure CL versus IL. The differences between the 2 tutorials are summarized in Table 2.

**Table 1:** Topics covered in the Electronic Health Information for Lifelong Learners tutorials used in this study.

Session number	Module number	Topic
Session 1	Module 1	Basic computer and internet terms
Session 2	Module 6	Introduction to MedlinePlus.gov and search for health topics on MedlinePlus.gov
Session 3	Module 7	Use of “Drugs and Supplement” on MedlinePlus.gov
Session 4	Module 8	How to find news, physicians, and hospitals and use multimedia on MedlinePlus.gov
Session 5	Module 8	How to find news, physicians, and hospitals and use multimedia on MedlinePlus.gov
Session 6	Module 9	How to identify the quality of health information on the internet

**Table 2:** Differences between the individualistic learning (IL) and collaborative learning (CL) tutorials.

	IL	CL
At the beginning of each tutorial	The tutorial provides visual instructions and the following audio instructions reminding the students to work independently: “During today’s lesson, you will work individually. This will include activities where we ask you to reflect on your own about what you have learnt. Please do not consult your fellow learners during this lesson. If you have any questions, consult the facilitator and they will answer any questions or concerns you might have.”	The tutorial provides visual instructions and the following audio instructions about how to work together: “During today’s lesson, you will work together with your group members to learn new skills and review the materials. Take turns completing the practice activities and moving the tutorials. If a group member has any difficulty, feel free to provide assistance. Think of yourself as a team that works together to help improve each other’s learning.”
Before performing each practice activity	The tutorial provides audio instructions to remind students to perform the practice activity individually, for example, “Now you are going to work individually to master the terms you just learnt.”	The tutorial provides audio instructions to remind students to work together to complete the practice activity, for example, “Now you are going to work together to practice what you just learnt” or “Take the next few minutes to work together to follow the instructions on the screen to open the quiz on germs and hygiene. Each partner should take turns operating the tutorial. If you encounter any difficulties, consult your group members for assistance. After each partner has a chance to practice, press the ‘Next’ button to continue.” Sometimes, the tutorial also provides visual instructions on how to practice together.
After completing each practice activity	The tutorial asks the students to try again or move on to the next practice.	The tutorial asks the students to restart the activity or try again until each group member has had a turn to practice.
At the end of each tutorial	The students review the lesson goals on their own.	Students review together the lesson goals covered in class. The following instruction is given: “Take the next few minutes to work together to review the following list of goals covered in today’s lesson. Click on the check box next to each goal to confirm that all group members are comfortable that they have mastered it. If anyone has any difficulty, work together to come to a solution and refer to the handout for further clarification.”
At the end of each learning goal in module 9	The students are reminded not to compare notes with anyone else.	In module 9, after students have accomplished each learning goal, they are asked to compare notes with their peers.

### Measures

We used both objective and subjective measures to assess the learning outcomes or serve as control variables. The measures and data collection times are summarized in Table 3.

**Table 3:** Measures used and time of measurement.

Category of measures and variable		Measure	Time^a^		
			1	2	3
**Objective learning outcome**					
	Knowledge acquisition	Objective tests of knowledge of computer components (eg, keyboard and mouse) and the web (eg, link and scroll bar). Computer knowledge and web knowledge were each measured using 10 items; each item was scored with 1 point if answered correctly and 0 points if answered incorrectly (scoring range 0-20).	✓	✓	✓
	Skill acquisition	3 procedural tests required participants to carry out specific tasks on networked computers: Basic computer and web operation: participants performed 12 basic operations on the computer (eg, open a web browser and go to a website). Each task was scored with 1 point if performed correctly or 0 points if done incorrectly (scoring range 0-12). Information seeking: participants received 4 scenarios in which they were asked to find information about specific health topics on the internet (eg, find at least two treatments for breast cancer). Each scenario was scored from 0 to 2 (0 if no relevant information was found, 1 if some but not all the required information was found, and 2 if all the required information was found; scoring range 0-8). Website evaluation: participants were asked to visit and evaluate the reliability of 8 health information websites. Evaluations were recorded as “Yes” if a website was reliable, “No” if a website was not reliable, and “Can’t decide” if the reliability of a website was unknown. Each website was scored with 1 if the evaluation result was correct or 0 if the evaluation result was incorrect or “Can’t decide” (scoring range 0-8).	✓	✓	✓
**eHealth literacy**					
	eHealth literacy efficacy	The 8-item eHealth literacy scale [14], which measures self-perceived skills and comfort with using IT for health information and decision-making. Items are scored on a 5-point Likert scale; higher scores indicate higher eHealth literacy efficacy (scoring range 8-40; Cronbach α=.89-.97, with good test-retest reliability) [15].	✓	✓	✓
**Previous experience**					
	Previous experience with computers and the internet	6 items, 4 of which measured the duration and frequency of previous computer and internet use. Example question: “How long have you been using a computer?” In total, 2 items measured previous computer class experience. Example: “Have you taken our computer class previously?”	✓		
**Control variables**					
	Familiarity with peers in the same class	If and how participants may be related to or familiar with others in the same experimental condition (eg, spouse, sibling, friend, or acquaintance)	✓		
	Standard health literacy test	S-TOFHLA^b^; 2 subscales with 36 questions; scoring range 0 to 36; Cronbach α=.97 (reading) and .68 (numeracy) [45]	✓		
	Demographic and health factors	Age, sex, education, health, race and ethnicity, income, and primary language	✓		
**Postintervention questionnaire**					
	Satisfaction	How would you evaluate your entire experience in this computer class? (“Extremely dissatisfied,” “Dissatisfied,” “Neither satisfied nor dissatisfied,” “Satisfied,” and “Extremely satisfied”)		✓	
	Whom did participants learn from?	During the past 4 weeks, while in class, from whom did you learn about computers? (“Peer student(s),” “Mostly the peer student(s),” “The tutorial and peer(s) equally,” “Mostly the tutorial,” and “The tutorial”)		✓	
	Interaction with peers	During the past 4 weeks, how much in-class interaction have you had with your peer(s)? (“None,” “A little,” “Some,” “A lot,” and “Extensive”)		✓	

^a^1: before the intervention; 2: after the intervention; 3: 6-month follow-up.

^b^S-TOFHLA: Short Test of Functional Health Literacy in Adults.

### Data Analysis

Trained graduate research assistants entered the data into SPSS (version 27.0; IBM Corp) for Windows, with the principal investigator monitoring data entry and cleaning by reviewing a random 10% of the data records. Before inferential analysis, the data were evaluated for accuracy, missing data, out-of-range values, and violation of the statistical assumptions. Background variables (demographics, previous experience, and language) were examined to detect potential differences between the 2 learning condition groups. Descriptive statistics were used to provide a statistical profile of the sample, with frequencies and percentages for categorical data and means and SDs for continuous data.

Mann-Whitney *U* tests and 2-tailed *t* tests were conducted to assess differences between participants who completed tests at all 3 time points and those who completed only the pretest measurement. A 3 (group composition based on previous computer experience: experienced, new, and mixed) × 2 (learning condition: IL vs CL) × 3 (time of measurement: pretest measurement, posttest measurement, and 6-month follow-up) mixed between-within univariate analysis of covariance was conducted on each of the dependent variables individually; the results for each outcome were of direct interest.

Using the 4 computer and internet experience variables, both factor analysis and cluster analysis were conducted. The factor analysis yielded one strong factor (eigenvalue 3.38 vs 0.38, 0.17, and 0.08), implying that the 4 variables are strongly related and could be used to define a continuum of computer and internet use that individuals have scores on. By contrast, the cluster analysis yielded a clean separation of 2-cluster solutions, which is characterized by mean comparisons that ranged between 1.28-1.95 (cluster 2) versus 4.38-5.08 (cluster 1). Cluster 1 is a group that has been using the computer and internet for 3 or more years and uses it at least weekly, if not more frequently. Cluster 2 is a group that has been using the computer or internet for less than a year and who typically accesses it less than once a month. This *computer and internet familiarity* dichotomous variable was used to further categorize the groups into 3 groups for group composition based on prior computer experience. The first grouping comprised of groups with <30% of participants being experienced (which we defined as the “new” user group composition in all subsequent analyses; 14/92, 15%). The second grouping comprised of groups with 30% to 70% of participants being experienced (the “mixed” group composition; 48/92, 52%). The third grouping comprised of groups with >70% of participants being experienced (the “experienced” group composition; 30/92, 33%).

The main outcome variables of interest were eHealth literacy efficacy, computer and web knowledge, and skill acquisition (3 measures: basic computer and web operation skills, information-seeking skills, and website evaluation skills). The Short Test of Functional Health Literacy in Adults (S-TOFHLA) was included as a covariate, as opposed to an outcome variable, as the intervention was not focused on the learning of outcomes measured by the S-TOFHLA. The S-TOFHLA was significantly correlated with all 5 outcomes, ranging from 0.11 (eHealth literacy efficacy) to 0.52 (computer and web knowledge).

To test the specific hypotheses of this study, we focused our analyses on the main effects of learning condition and on interactions between learning condition and previous computer experience. The main effects of previous computer experience were not a major focus as it is already well documented that previous computer experience is predictive of older adults’ computer adoption and use [46-48]. Given the expected interactions, tests of simple effects within specific levels of the design were likely [49], such as those assessing the differential impact of learning condition within the experienced, new, and mixed levels of previous computer experience.

The models were conducted with and without control variables. A consistent pattern of seeing no differences with and without control variables was observed. The control variables included age, sex, education, health status, race and ethnicity, income, and primary language. The inclusion of these control variables could increase the statistical power of the design given that the variance in outcomes would likely be due, in part, to variability in one or more of these variables. Tests of the main effects were conducted in the absence of interactions involving the main effect variables. The resulting effect size estimates were calculated to compare the magnitude of change for the different types of dependent variables and between time points using *η*_p_^2^ [50].

## Results

### Participants

Participants’ demographics and other background information are summarized in Table 4. Participants were randomized into the IL (233/466, 50%) or CL (233/466, 50%) group. Chi-square and *t* tests showed no significant differences in baseline characteristics between the IL and CL groups except in English as participants’ primary language (*χ*^2^_1_=5.6; *P*=.02); English was the primary language of more participants in the IL group than in the CL group (211/233, 90.6% vs 194/233, 83.3%). A total of 85.4% (398/466) of the original sample completed the postintervention assessment, and 41% (191/466) completed the 6-month follow-up assessment.

**Table 4:** Participant characteristics (N=466).

		All, n (%)	IL^a^ group (n=233), n (%)	CL^b^ group (n=233), n (%)	*P* value for chi-square or *t* test	
Sex (female)		312 (67)	154 (66.1)	158 (67.8)	.69	
**Race and ethnicity**						.10
	African American	188 (40.3)	81 (34.8)	107 (45.9)		
	White	153 (32.8)	84 (36.1)	69 (29.6)		
	Latino	88 (18.9)	49 (21)	39 (16.7)		
	Other	37 (7.9)	19 (8.2)	18 (7.7)		
**Education**						.99
	Lower than high school	52 (11.2)	27 (11.6)	25 (10.7)		
	High school	116 (24.9)	58 (24.9)	58 (24.9)		
	Some college	159 (34.1)	80 (34.3)	79 (33.9)		
	Bachelor’s degree or higher	138 (29.6)	68 (29.2)	70 (30)		
**Yearly household income (US $)**						.12
	<20,000	163 (35)	88 (37.8)	75 (32.2)		
	20,000-29,000	86 (18.5)	38 (16.3)	48 (20.6)		
	30,000-39,000	48 (10.3)	30 (12.9)	18 (7.7)		
	40,000-99,000	91 (19.5)	37 (15.9)	54 (23.2)		
	≥100,000	9 (1.9)	4 (1.7)	5 (2.1)		
Native English speaker (yes)		405 (86.9)	211 (90.6)	194 (83.3)	.02	
Health status (excellent and very good)		123 (26.4)	65 (27.9)	58 (24.9)	.46	
**Frequency of computer use**						.84
	Never	151 (32.4)	75 (32.2)	76 (32.6)		
	Less than once a month	54 (11.6)	29 (12.4)	25 (10.7)		
	More than once a month	35 (7.5)	18 (7.7)	17 (7.3)		
	Once a week	47 (10.1)	20 (8.6)	27 (11.6)		
	Every 2-3 days	86 (18.5)	41 (17.6)	45 (19.3)		
	Every day	93 (20)	50 (21.5)	43 (18.5)		
**Length of computer use (years)**						.86
	Never	130 (27.9)	67 (28.8)	63 (27)		
	<1	91 (19.5)	42 (18)	49 (21)		
	1-3	51 (10.9)	27 (11.6)	24 (10.3)		
	3-5	32 (6.9)	18 (7.7)	14 (6)		
	5-10	64 (13.7)	29 (12.4)	35 (15)		
	>10	96 (20.6)	49 (21)	47 (20.2)		
**Frequency of internet use**						.98
	Never	196 (42.1)	98 (42.1)	98 (42.1)		
	Less than once a month	53 (11.4)	27 (11.6)	26 (11.2)		
	More than once a month	27 (5.8)	13 (5.6)	14 (6)		
	Once a week	36 (7.7)	17 (7.3)	19 (8.2)		
	Every 2-3 days	72 (15.5)	34 (14.6)	38 (16.3)		
	Every day	81 (17.4)	43 (18.5)	38 (16.3)		
**Length of internet use (years)**						.84
	Never	185 (39.7)	93 (39.9)	92 (39.5)		
	<1	78 (16.7)	35 (15)	43 (18.5)		
	1-3	50 (10.7)	27 (11.6)	23 (9.9)		
	3-5	35 (7.5)	18 (7.7)	17 (7.3)		
	5-10	62 (13.3)	29 (12.4)	17 (7.3)		
	>10	56 (12)	31 (13.3)	33 (14.2)		
Familiar with other participants (yes)		126 (27)	68 (29.2)	58 (24.9)	.31	

^a^IL: individualistic learning.

^b^CL: collaborative learning.

### Participants Who Completed All 3 Time Points Versus Those Who Did Not

In *t* tests and Mann-Whitney *U* tests, no significant differences were found for baseline age (*P*=.623), education (*P*=.052), health (*P*=.090), language (*P*=.705), income (*P*=.893), computer and web knowledge (*P*=.453), basic computer and web operation (*P*=.731), and website evaluation (*P*=.929) between participants who completed all 3 time points and those who completed only the pretest measurement. There were no statistically significant differences in dropout rates between the IL and CL groups from baseline to postintervention measurement (*P*=.660) and from baseline to 6-month follow-up (*P*=.778).

Significant differences were found between those who completed all 3 time points and those who did not for sex (*P*=.009), race and ethnicity (*P*<.001), baseline computer use length (*P*=.001), internet use length (*P*<.001), computer use frequency (*P*=.004), internet use frequency (*P*<.001), eHealth literacy efficacy (*P*<.001), health literacy (*P*=.04), and information-seeking skills (*P*=.02). In comparison with participants who completed only the pretest measurement, there was a higher proportion of women (62% vs 74%), a lower proportion of African American individuals (51% vs 26%), and a higher proportion of those who reported more frequent and longer length of computer (17% vs 25%) or internet use (8% vs 17%) at baseline among participants who completed all 3 time points. Participants who completed all 3 time points also reported significantly higher scores on the eHealth literacy efficacy scale (mean difference=1.56), S-TOFHLA (mean difference=2.52), and information-seeking skill test (mean difference=1.27) at baseline.

### Tests of Hypotheses

Examination of general linear models revealed 1 statistically significant model of interest (Table 5). The model with basic computer and web operation skills as the outcome resulted in a significant interaction that supported hypothesis 3. There was a significant interaction between learning condition and previous computer experience (*F*_2,55_=3.69; *P*=.03). Simple effects were examined to decompose interaction results. Specifically, from postintervention measurement to 6-month follow-up, within the IL group, on average, being in a group with little or no previous computer experience (mean −0.59, SE 0.54) was more beneficial for retaining computer skills than being in a group with medium previous computer experience (mean −1.06, SE 0.25) or high previous computer experience (mean −1.65, SE 0.35). In comparison, in the CL group, on average, being in a group with high previous computer experience (mean −0.60, SE 0.35) was more beneficial for retaining computer skills than being in a group with medium previous computer experience (mean −0.64, SE 0.21) or little to no previous computer experience (mean −2.40, SE 0.82). Hypothesis 3 was partially supported; that is, the effects of CL were better maintained than those of IL for individuals in certain groups. Specifically, *for people with little to no previous experience, it may be better to learn individually, whereas for people with more previous experience, it may be better to learn collaboratively*. These results were specific to computer and web operation skills and maintenance of those skills at 6 months after the intervention.

No statistically significant differences were found in models examining the interactions between learning condition and previous computer experience for each of the following outcome measures: eHealth literacy efficacy, computer and web knowledge, information-seeking skills, and website evaluation skills (results not shown; available from the authors upon request). Hypotheses 1 and 2 were not supported.

**Table 5:** General linear model results for retaining basic computer and web operation skills from postintervention measurement to 6-month follow-up.

	Basic computer and web operation	
	Wald *F* test (*df*)	*P* value^a^
IL^b^ or CL^c^ learning condition	0.09 (1)	.77
Computer familiarity	6.44 (1)	.01
Computer experience grouping	0.94 (2)	.40
IL or CL × computer familiarity	2.14 (1)	.15
IL or CL × previous experience grouping	3.69 (2)	.03
Computer familiarity × experience grouping	0.42 (2)	.66
IL or CL × computer familiarity × experience grouping	2.04 (2)	.14

^a^α=.05.

^b^IL: individualistic learning.

^c^CL: collaborative learning.

### Main Effects

Univariate repeated-measure analyses revealed statistically significant differences between pretest measurement, posttest measurement, and 6-month follow-up for all 5 outcome measures (Table 6).

Follow-up comparison tests for these 5 outcome measures showed statistically significant improvements in mean scores from pre- to posttest measurement (*P*<.001 for all 5 cases). Follow-up comparison tests also showed statistically significant decreases in mean scores from posttest measurement to 6-month follow-up for 3 of the 5 outcome measures: eHealth literacy efficacy, computer and web knowledge, and basic computer and web operation skills (*P*<.001 for all 3 cases). There was no statistically significant difference in mean scores from posttest measurement to 6-month follow-up for the remaining 2 outcome measures—website evaluation (*P*=.774) and information-seeking skills (*P*=.365).

**Table 6:** Means, *F* test results, and effect sizes.

Dependent variable	Pretest measurement, mean (SD)	Posttest measurement, mean (SD)	6-month follow-up, mean (SD)	*F* test (*df*)^a^	*η*_p_^2^
eHealth literacy efficacy	19.86 (8.08)	33.37 (4.68)	32.06 (5.93)	373.82 (2)	0.676
Computer and web knowledge	12.59 (4.69)	16.44 (3.08)	15.66 (3.30)	89.60 (2)	0.334
Basic computer and web operation	6.29 (3.75)	10.17 (2.10)	9.44 (2.62)	167.92 (2)	0.484
Information-seeking skills	2.18 (2.63)	3.69 (2.63)	4.32 (2.56)	52.92 (2)	0.228
Website evaluation skills	4.02 (2.00)	5.04 (1.89)	5.24 (2.05)	35.80 (2)	0.167

^a^*P* value for all *F* test values is <.001.

### Postintervention Questions

There were no significant differences in participants’ satisfaction between the CL (100% satisfaction) and IL (99% satisfaction) groups (N=382, *χ^2^*_1_=1.1; *P*=.29). Overall, participants in both groups had satisfactory experiences with the intervention.

There were significant differences between the CL and IL groups for learning with peers versus tutorials (N=382, *χ^2^*_4_=29.2; *P*<.001) and for the amount of in-class interaction with peers (N=382, *χ^2^*_4_=84.3; *P*<.001). Demonstrating validity, individuals in the CL group reported learning from a combination of peers and the tutorial, whereas individuals in the IL group reported learning “exclusively” from the tutorial. Similarly, individuals in the IL group reported low interaction with peers, whereas individuals in the CL group reported high interaction with peers.

## Discussion

### Principal Findings

Older adults are less likely than younger adults to use the internet for tasks such as receiving test results, renewing prescriptions, and scheduling appointments, in part because of a low level of digital competence [51]. The COVID-19 pandemic has made accessing health information and services on the web a near necessity [52,53], exacerbating the need for eHealth literacy. Effective interventions are much needed to ensure the digital inclusion of older adults during and after the pandemic. This study’s principal findings are as follows: (1) there are no major differences in older adults’ eHealth literacy learning with regard to learning collaboratively versus individually when measured immediately after the intervention; (2) however, to maintain long-term benefits, it may be best to learn collaboratively with others that have similar previous computer experience; (3) regardless of the IL or CL method, this intervention was effective for increasing eHealth literacy in older adults; and (4) conducting periodic follow-up training (ie, booster sessions) may be important for improving the maintenance of gains over time. It is important to note that our sample included a large proportion of African American (188/466, 40.3%) and Latino (88/466, 18.9%) participants. The literature on eHealth interventions over the past 2 decades documents the lack of studies with racial and ethnic minority samples as well as the continued need to include these groups in studies [54-57]. This study’s inclusion of a substantial proportion of participants from racial and ethnic minorities strengthens the evidence that our eHiLL intervention works for older adults from diverse groups.

### CL Versus IL

In this study, we compared the impact of CL versus IL on older adults’ learning of eHealth literacy and digital skills. Our data did not provide support for hypotheses 1 and 2. However, hypothesis 3 was partially supported. For participants in a group with medium or high previous computer experience, learning was maintained better at the 6-month follow-up in the CL condition, whereas in the IL condition, learning was maintained better for those in a group with low previous computer experience. Thus, previous computer experience may mediate the relationship between learning outcomes and learning methods.

We did not find any statistically significant differences from pre- to posttest measurement in the effects of CL versus IL on any of the outcome measures. This finding aligns with a previous study that used an earlier iteration of the intervention to test IL and CL [20]. The 5 principles of CL might shed light on why [36]. These principles are as follows: (1) to ensure that students understand that their scores are dependent on both their individual and group members’ performances (eg, by giving bonus points to each student if all members of the group score at a certain percentage or higher on a test); (2) to structure individual accountability so that each student’s individual contribution is assessed (eg, by giving individual tests, having each student explain their contribution to the group, or observing group interactions and documenting each student’s contributions); (3) to ensure that students help, assist, support, encourage, and praise one another’s learning efforts through face-to-face interactions; (4) to ensure that students have needed social skills (eg, communication and leadership) and use them properly in the group; and (5) to ensure that students have adequate time to engage in group interactions, reflect on what works and what does not, and make decisions about what actions to continue or change.

However, these principles have been developed for formal educational settings, and they are less applicable to informal settings [24,36]. Therefore, in this study, not all the principles were included. For example, the literature suggests that it is important to build dependency and accountability, which would work in formal educational settings [36]. In this study’s context, individual success was not designed to be dependent on group success. Furthermore, this study, by nature, could not hold individual group members accountable for group success in any formal way. Each participant’s individual contribution was not formally assessed, but it is plausible that having each participant explain their contribution to the group or observing group interactions and documenting participants’ contributions might create a stronger sense of accountability within the group. Future research should assess creative ways to ensure dependency and accountability among older adult learners in informal settings.

The literature has found CL to work in informal settings with older adults [26], although this has not been supported with regard to computer training from pre- to posttest measurement [20,21,34]. Previous eHiLL studies [20,21,34] did not conduct follow-ups, so there was no evidence on the longitudinal effects of CL. In this study, which included a follow-up, CL did work better under certain conditions over the longer term.

Computer learning is more challenging than other subjects in informal learning [58]. An additional challenge is that CL research generally does not provide detailed instructions to ensure collaboration [59]. Therefore, in this study, we provided more detailed instructions for collaboration as the participants progressed through the modules. The participants’ responses indicated that they did learn either collaboratively or individually in accordance with their group assignment; it is unlikely that our CL strategies were insufficient in soliciting CL.

### Group Composition

Similar to hypothesis 1, hypothesis 2 was rejected, and no statistically significant differences were found between either learning condition and group composition. However, the partial support for hypothesis 3 may provide some insights into how group composition may affect learning outcomes over the longer term. Group composition appears to matter depending on the characteristic used to group participants. In this study, there were no differences between the CL and IL groups with regard to the familiarity of the participants within their groups. However, the literature has documented familiarity with group partners as a factor that may contribute to increased collaboration [40,41,60]. The relative benefits of CL versus IL among older adults—with familiar or unfamiliar partners—require further examination.

Our findings do indicate that grouping by previous computer experience may be particularly important for older adults’ learning over the longer term. This study’s findings are complemented by those from an earlier study with previous versions of the tutorial [20]. On the basis of information from the previous study, the differences in participants’ previous computer experience might have at least partially affected their learning experience and outcomes. Xie [20] found that more experienced learners sometimes became frustrated and felt that they were not making the best use of their time when an instructor had to stop frequently to help less experienced peers keep up with basic procedures (eg, manipulating a mouse). The opposite happened as well: less experienced learners sometimes became embarrassed and frustrated and at times were intimidated by more experienced peers. These observations, in addition to guidance from the literature [43], support the change in this study to separate older adults into different groups based on their previous computer experience. This study’s findings suggest that CL may be most beneficial for more experienced older adults when they are grouped with others who have similar levels of previous computer experience. Perhaps, when group members have already obtained sufficient previous experience, they can use their collective previous experience to learn from each other [61]. In comparison, CL groups with low collective previous experience may not have sufficient combined skills and knowledge to progress effectively. A recent systematic review also suggests that knowledge level and experience seems to be the most suitable and important attribute to form educational groups because of its effects on group outcomes [62]. Thus, IL may be better over the long term for older adult learners with low previous computer experience as each individual can move at a comfortable pace and may not be embarrassed to ask for help.

### Overall Impact of the eHealth Intervention

In this study, we examined the intervention’s effects on 5 outcomes: eHealth literacy efficacy, computer and web knowledge, basic computer and web operation, information-seeking skills, and website evaluation skills. Overall, the results show statistically significant improvements from before to after the intervention for all 5 outcomes (*P*<.001 in all cases). Thus, the intervention, with CL or IL, is effective in improving older adults’ eHealth literacy. Effect sizes ranged from 0.167 to 0.676, suggesting that the magnitude of improvement was large for all 5 outcomes (according to the general guidelines used to interpret values for effect sizes: 0.01=small effect size, 0.06=medium effect size, and ≥0.14=large effect size) [50]. These results align with previous eHiLL studies, which included 3 of the same outcome variables (computer and web knowledge, computer and web skills, and eHealth literacy) also with large effect sizes [20,21,33,34]. The consistently large effects of the intervention in different populations and contexts highlight the potential generalizability of the intervention to improve older adults’ eHealth literacy.

### Longitudinal Effects

In this study, we included a follow-up at 6 months to assess how well improvements were retained. Overall, there was a decrease in scores from postintervention measurement to 6-month follow-up, with statistically significant decreases for 3 of the 5 outcomes (eHealth literacy efficacy, computer and web knowledge, and basic computer and web operation; *P*<.001 for all 3 cases). The decreases in information-seeking skills and website evaluation were not statistically significant. eHealth literacy is multidimensional, and some of its components may be retained better than others over time. However, it is possible that a worse long-term impact might have been observed overall if a longer follow-up was used, such as 1 year. A recent study assessing cognitive training for older adults found that little or no benefit remained 1 year after intensive initial training [63]. This study’s findings suggest a need for resources to provide continuous training or periodic boosting given that benefits gained from pre- to posttest measurement dropped after 6 months. A study [64] assessing cognitive ability training with older adults that included long-term booster training (11 and 35 months after the intervention) and long-term follow-up tests (1, 3, and 5 years) found that the effects of the intervention were still present 5 years later. Further research is needed to understand how often such “booster” training is needed for this intervention to maximize resources (eg, every 1, 2, or 3 months). Another promising training component to consider for observing long-term effects might be frequent testing, which may lead to a practice-retrieval effect—there is some evidence suggesting that more frequent testing during an intervention phase is associated with long-term skill retention [65].

In addition to including the follow-up at 6 months, this study also addressed other limitations of previous eHiLL studies. We included a large sample size, a more even baseline group composition and group size because of randomization, and consistency of instructors. In earlier eHiLL studies, because of limited funding, it was not feasible to control for variation in instructors. Earlier eHiLL studies used many graduate students as instructors for the training classes, who were recruited through various mechanisms—some were part-time research assistants, some received course credits, and some were simply volunteers. These graduate students’ enthusiasm speaks to the sustainability of the training program, but from the point of view of an intervention study, individual differences among such instructors (eg, personality, teaching style, experience, time commitment, and incentive) likely introduced unnecessary confounding variation into the previous studies. Therefore, in this study, we provided full-time support for a few instructors to minimize the potential impact of this factor. In short, compared with the earlier studies, this study provides stronger support for the effectiveness of the intervention overall and its various components. This evidence should enable other researchers to replicate this work using other samples, settings, learning conditions, or delivery methods.

### Strengths and Limitations

First, this study’s large sample size ensured sufficient statistical power for the findings; however, the sample may not be representative of the older adult population in general. Second, differential dropout by race and ethnicity, sex, and computer experience suggests that additional tailoring is needed to promote better adherence. For example, He et al [66] found evidence that machine learning–based approaches provided with individual characteristics and previous intervention data can provide useful information for predicting adherence, providing initial clues as to who to target with adherence support strategies and when to provide support. Further assessment of these types of innovations will be critical to strengthen an intervention’s ability to support those at risk of poor adherence. Other researchers should replicate this study in other communities with different samples of older adults, which would help further strengthen the eHiLL intervention’s generalizability.

Third, an inevitable limitation of any technology-related intervention is that technology evolves rapidly, rendering some intervention components (and corresponding outcome measures) outdated. Continuous updating of intervention components and outcome measures will be necessary for future interventions (eg, to be based on mobile devices and apps and voice-based web search enabled by new technology). Our findings suggest that eHealth literacy is multidimensional and that some of its components (eg, the ability to search for relevant information on the web to solve specific tasks, as measured in our information-seeking skill testing, and assess the quality of health information on websites, as measured in our website evaluation test) might be better retained than others (eg, eHealth literacy efficacy, computer and web knowledge, and basic computer and web operation) over time. This phenomenon is worth future systematic investigation, which might lead to the development of more granular interventions targeting specific dimensions or components of eHealth literacy.

### Implications and Directions for Future Research

Given the context of this work in the larger program of work that has been carried out over a decade, there is strong evidence to suggest that older adults can increase their eHealth literacy via various learning conditions. This is important as increasing eHealth literacy for older adults can have substantial positive impacts on their health management in several ways. For example, those who are eHealth literate can take advantage of the many technologies that allow health care providers to monitor one’s health remotely in real time. Older adults with diabetes may upload food logs, blood sugar levels, and drugs taken that providers can check daily and provide feedback on [67]. Also relevant for older adults are tools that can detect changes in daily activities, such as falls, and devices that send notifications to remind one to exercise or take drugs [67]. These technologies are only useful if one has the eHealth literacy to use them.

In addition, being able to incorporate eHealth into the management of their health can possibly help reduce other stressors. For example, knowing how to navigate a web-based health portal can help save time by allowing older adults to communicate with their providers through web-based messages as opposed to waiting on the phone just to pass their message along to someone who is not their physician. This could possibly reduce the stress associated with missing a phone call from their physician’s office, knowing that they can check their message at any time as soon as it is available. Similarly, older adults can save time by making an appointment with their physician on the web. As telehealth becomes more common, when appropriate, older adults can take appointments in the comfort of their own homes, which can help reduce stress related to finding a date and time that works best for them to find transportation. eHealth has the potential to increase the health and well-being of older adults, and increasing eHealth literacy is one step toward helping them do so.

Further research should also examine how often booster training may be needed to maximize resources. Scalability should be of interest for future studies to assess the mass-scale impact this intervention can have. Future studies are being planned to assess the intervention’s effectiveness with remote learning or hybrid modalities that may be better suited to a world coping with the COVID-19 pandemic. The finding that the intervention is effective with remote learning modalities will help strengthen the evidence that this intervention has the potential to be scalable at a national level. Finally, in this study, previous-experience group composition was a key factor; however, future research could assess if there are other, more effective group composition possibilities, such as groups based on self-assessed technology proficiency (ie, computer, mobile device, and networking proficiency). Tools assessing these proficiency levels [68-70] can be incorporated to assess whether composing groups based on overall high and low technology proficiency leads to more effective training, as suggested by the interactions found between group composition and learning condition.

## Appendix

Multimedia Appendix 1 Instructions at the beginning of the individualistic learning (IL) tutorial.

Multimedia Appendix 2 Instructions at the beginning of the collaborative learning (CL) tutorial.

Multimedia Appendix 3 Example of Instructions before practice activities in the collaborative learning (CL) tutorial (Step 1, Module 2).

Multimedia Appendix 4 Instructions before practice activities in the collaborative learning (CL) tutorial (Step 3, Module 2).

Multimedia Appendix 5 Instructions after completing each practice activity in the collaborative learning (CL) tutorial.

Multimedia Appendix 6 Instructions after completing each learning goal in the collaborative learning (CL) tutorial (Module 9).

## Abbreviations

CL: collaborative learning
eHiLL: Electronic Health Information for Lifelong Learners
IL: individualistic learning
NIH: National Institutes of Health
S-TOFHLA: Short Test of Functional Health Literacy in Adults

## Data Availability

The data sets generated and analyzed during this study are available from the corresponding author upon reasonable request.
