# Supplementary figures and images for: Individualistic Versus Collaborative Learning in an eHealth Literacy Intervention for Older Adults: Quasi-Experimental Study
Source: JMIR Aging. 2023 Feb 9;6:e41809. doi: 10.2196/41809 (PMC9951071; doi:10.2196/41809)

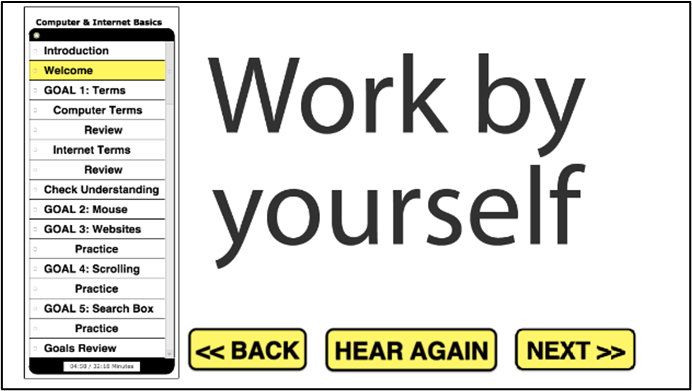

Supplement: Multimedia Appendix 1 [file aging_v6i1e41809_app1.png]

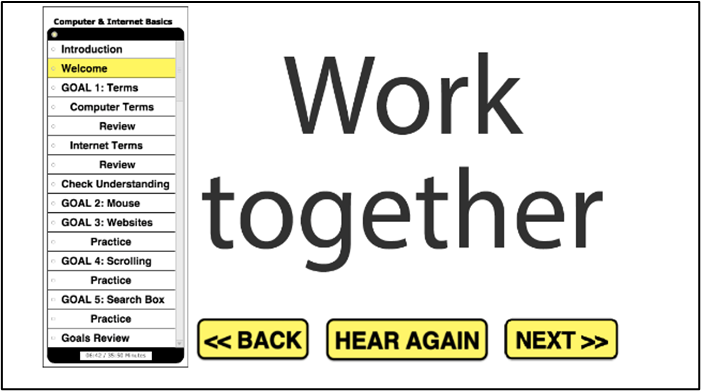

Supplement: Multimedia Appendix 2 [file aging_v6i1e41809_app2.png]

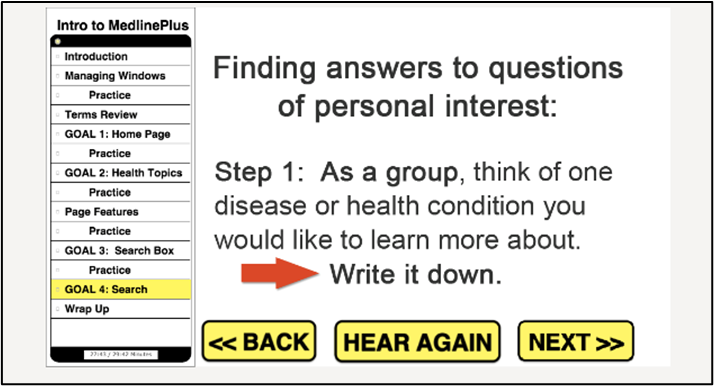

Supplement: Multimedia Appendix 3 [file aging_v6i1e41809_app3.png]

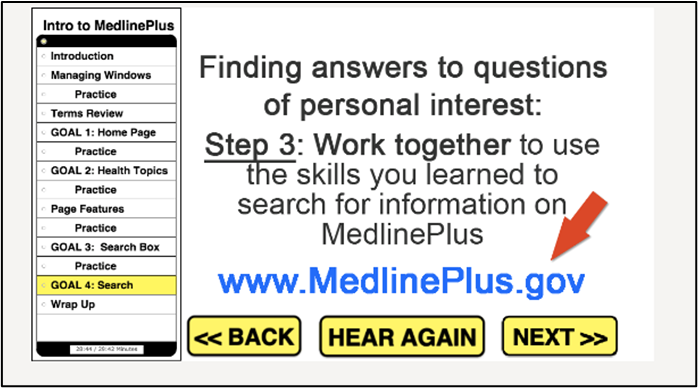

Supplement: Multimedia Appendix 4 [file aging_v6i1e41809_app4.png]

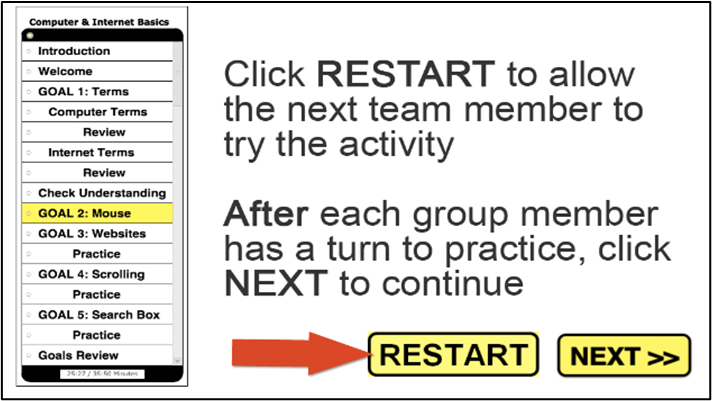

Supplement: Multimedia Appendix 5 [file aging_v6i1e41809_app5.png]

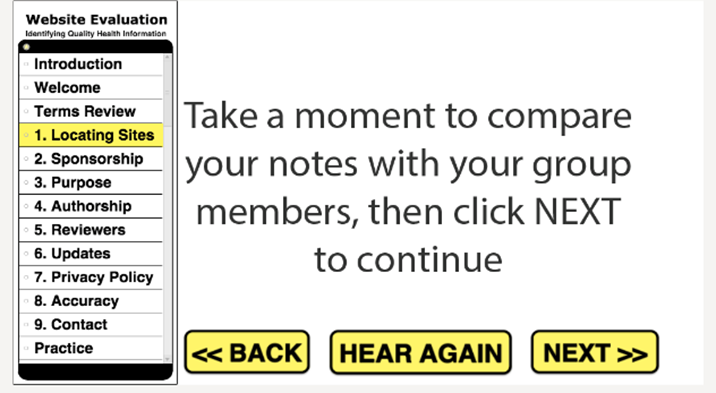

Supplement: Multimedia Appendix 6 [file aging_v6i1e41809_app6.png]
